# Excess Properties, FT-IR Spectral Analysis, and CO_2_ Absorption Performance of Monoethanolamine with Diethylene Glycol Monoethyl Ether or Methyldiethanolamine Binary Solutions

**DOI:** 10.3390/molecules30071523

**Published:** 2025-03-29

**Authors:** Maria Magdalena Naum, Mihaela Neagu, Vasile Dumitrescu

**Affiliations:** 1 Chemistry Department, Petroleum-Gas University of Ploiesti, 100680 Ploiesti, Romania; vdumi@upg-ploiesti.ro; 2 Petroleum Refining and Environmental Engineering Department, Petroleum-Gas University of Ploiesti, 100680 Ploiesti, Romania

**Keywords:** density, viscosity, excess properties, FT-IR spectra, CO_2_ absorption

## Abstract

In this study, densities and viscosities of the binary solutions of monoethanolamine with diethylene glycol monoethyl ether or methyldiethanolamine were determined at 293.15, 298.15, and 303.15 K and *p* = 100.5 kPa. The experimental density data were tested with different equations as a function of composition (Belda and Herraez equations) and as a function of temperature and composition (Emmerling et al. and Gonzalez-Olmos–Iglesias equations). The results show that the Herraez and Emmerling et al. equations best correlate the experimental data. The experimental values of viscosity were tested with different models based on one, two, three, or four parameters. The values of excess molar volume (*V*^E^), viscosity deviation (Δ*η*), and excess Gibbs energy (ΔG*^E^) were calculated from the experimental values and were fitted to the polynomial equations. The values of the excess molar volume are negative for both systems, while positive values were obtained for the viscosity deviation and excess Gibbs activation energy. The values of thermodynamic functions of activation of viscous flow were determined and discussed. The Fourier transform infrared spectroscopy (FT-IR) spectra of the binary solutions analyzed in this study enabled the understanding of the interactions among the molecules in these solutions. In addition, the CO_2_ absorption capacity of the binary solutions of monoethanolamine with diethylene glycol monoethyl ether or methyldiethanolamine was determined experimentally.

## 1. Introduction

In recent times, the development of industrialization and globalization has led to increased energy consumption and especially the amount of CO_2_ as the main component of greenhouse gases resulting from the burning of fossil fuels, which has aggravated global climate changes [1]. Consequently, CO_2_ capture and storage (CCS) is a crucial technology for addressing the warming global climate, significantly contributing to reducing the greenhouse effect, and providing low-carbon thermal power plants [2,3,4]. In the industrial sector, technologies for capturing CO_2_ primarily consist of absorption, adsorption, low-temperature separation, membrane separation, biofixation, and oxygen-fuel combustion [5,6]. Among them, the adsorption technique mainly employs porous substances for CO_2_ capture, necessitating a high partial pressure for uptake and elevated temperature for desorption. The primary obstacle it encounters is high energy consumption [7,8]. In the meantime, the selectivity and stability of the membrane restrict membrane separation, while the stringent preparation process and slow processing rate of biological methods limit their use [9,10]. Nonetheless, chemical absorption is commonly utilized because of its benefits of high absorption capability and efficiency [11]. Generally, chemical absorption utilizes organic amines as a solvent that can interact with CO_2_ to produce salts, effectively capturing CO_2_ [6,11,12,13].

Of the chemical absorbents used for solvent-based absorption, aqueous monoethanolamine (MEA), particularly 30 mass% MEA, is the most commonly utilized solvent because of its low cost and high reactivity with CO_2_ [14]. Nonetheless, the desorption process demands a substantial amount of energy and needs considerable energy for solvent recovery, which is the primary disadvantage hindering its widespread implementation worldwide. There is significant concern regarding aqueous absorbents, as utilizing water as a solvent presents considerable disadvantages, including the higher heat capacity and vaporization enthalpy of water compared to organic solvents [15,16].

Binary and ternary mixtures of solvents formed by amines and other organic compounds such as alcohols, ethers, and glycols have contributed to the development of non-aqueous absorbents for CO_2_ absorption, as an alternative to aqueous solutions of amines. The use of these organic solvents contributes to saving regeneration energy and significantly reducing degradation and corrosivity [17].

Zhai et al. [18], Tan et al. [19], and Guo et al. [17] investigated different nonaqueous systems of MEA with some organic solvents, such as diethylenetriamine, triethylene glycol, and 2-alcoxyethanols (2-ethoxyethanol and 2-butoxyethanol). Zang et al. [20], Chai et al. [21], and Cao et al. [22] studied systems of ethylenediamine with diethylene glycol monomethyl ether, *n*-propanol/isopropanol, and methoxyethanol. Other organic amines (i.e., 1,2-propylenediamine, diethylenetriamine, N-methyldiethanolamine, and 1,2-propanediamine) have also been studied in mixtures with various organic solvents such as alcohols, ethers, and glycols [23,24,25,26].

The physical characteristics of amine solvents are beneficial in multiple fields such as process design, equipment sizing, mathematical modeling, and simulations. Density information is valuable for assessing the physical solubility of CO_2_ in solvents, mass transfer, and solvent kinetics. Viscosity information is essential for determining diffusivity using a modified Stoke–Einstein equation [27], which is necessary for evaluating mass transfer and kinetic properties [28]. Moreover, this information is necessary for developing thermodynamic models and to identify model parameters [29].

In this paper, the densities and viscosities of diethylene glycol monoethyl ether (DEGMEE) + monoethanolamine (MEA) and methyldiethanolamine (MDEA) + monoethanolamine (MEA) solutions were determined at 293.15, 298.15, and 303.15 K and *p* = 100.5 kPa, over the entire concentration range. The excess properties (*V*^E^, ∆η, and Δ*G*^#E^) were calculated and correlated with the polynomial equations. The activation thermodynamic functions of viscous flow were computed and discussed. FT-IR analysis of binary solutions was performed to explore the interactions between the molecules. To our knowledge, these systems have not been studied before.

## 2. Results and Discussion

### 2.1. Density and Viscosity

The experimental results of densities and viscosities for pure MEA, DEGMEE, and MDEA are in good agreement with the values from literature and are listed in Table 1. The density measurements for MEA vary by as much as 0.13% across different studies [30,31,32,33,34,35], while for DEGMEE, the variation is under 0.14% based on various sources [36,37,38,39]. For MDEA, the experimental density values differ by a maximum of 0.2% from those in the literature [25,40,41,42,43]. The viscosity values for MEA obtained from experiments exhibit variations of up to 1.7% when compared to those reported in sources [30,31,44,45], whereas those for DEGMEE show a maximum difference of 1.2% in comparison with the literature source [36]. For MDEA, the difference between experimental viscosity values and those in the literature [40,41,42,46,47,48] is a maximum of 1.6%. The experimental densities and viscosities for the mixtures are listed in Table 2, while the graphs are shown in Figures S1–S4. For the DEGMEE + MEA system, the density and viscosity of solutions increased as the concentration of amine increased and decreased as the temperature increased at a same concentration. For the MDEA + MEA system, the density and viscosity of solutions increased as the concentration of MDEA increased and decreased as the temperature increased. As the temperature increases, the molecular kinetic energy is higher; thus, the density and viscosity of the solution decreases [22].

The experimental density values were correlated as a function of composition with the Belda [49] (Equation (1)) and Herraez [50] (Equation (2)) equations, and as a function of temperature and composition with the Emmerling et al. [51] (Equation (3)) and Gonzalez-Olmos–Iglesias [52] (Equation (4)) equations.

(1) $$\rho =\rho_{2}+\left(\rho_{1}-\rho_{2}\right)x_{1}\left(\frac{1+m_{1}\left(1-x_{1}\right)}{1+m_{2}\left(1-x_{1}\right)}\right)$$

(2) $$\rho =\rho_{2}+\left(\rho_{1}-\rho_{2}\right)x_{1}^{A+Bx_{1}+Cx_{1}^{2}}$$

(3) $$\rho =x_{1}\rho_{1}+x_{2}\rho_{2}+x_{1}x_{2}\left[\begin{array}{c} P_{1}+P_{2}T+P_{3}T^{2}+\left(P_{4}+P_{5}T+P_{6}T^{2}\right)\left(x_{1}-x_{2}\right)+\\ \left(P_{7}+P_{8}T+P_{9}T^{2}\right){\left(x_{1}-x_{2}\right)}^{2} \end{array}\right]$$

(4) $$\rho =\sum_{i=0}^{2}Z_{i}x^{i}$$

In Equation (3), the density of each component varies with temperature according to the equation:

(5) $$\rho_{i}=A_{i}+B_{i}T+C_{i}T^{2}\;\left(i=1,\;2\right)$$

The coefficients *Z*_i_ in Equation (4) are calculated using the following equation:

(6) $$Z_{i}=\sum_{j=0}^{2}Z_{ij}T^{i}$$

The adjustable parameters were calculated with the Levenberg–Marquardt algorithm [53], and their values are presented in Tables S1 and S2. The validity of these equations was verified using the standard deviation calculated with Equation (7), and the values are also presented in Tables S1 and S2.

(7) $$\sigma ={\left[\frac{\sum {\left(X_{exp}-X_{calc}\right)}^{2}}{m-n}\right]}^{1/2}$$

Here, *X* denotes the values of the given properties, and *m* and *n* signify the number of experimental values and fitting parameters, respectively.

From these results, it is observed that the Herraez equation correlated better the density values as a function of composition compared to the Belda equation, and the Emmerling et al. equation is more efficient to correlate the density as a function of temperature and concentration.

The Grunberg–Nissan [54] (one parameter, Equation (8)), Heric–Brewer [55] (two parameters, Equation (9)), four-body McAllister [56] (three parameters, Equation (10)), and Jouyban–Acree [57,58] (four parameters, Equation (11)) models were tested to verify the dependence of viscosity as a function of concentration.

(8) $$ln\eta =x_{1}ln\eta_{1}+x_{2}ln\eta_{2}+x_{1}x_{2}d$$

(9) $$ln\eta =x_{1}ln\eta_{1}+x_{2}ln\eta_{2}+x_{1}lnM_{1}+x_{2}lnM_{2}-ln\left(x_{1}M_{1}+x_{2}M_{2}\right)+x_{1}x_{2}\left[\alpha_{12}+\alpha_{21}\left(x_{1}-x_{2}\right)\right]$$

(10) $$ln\eta =x_{1}^{4}ln\eta_{1}+4x_{1}^{3}x_{2}ln\eta_{1112}+6x_{1}^{2}x_{2}^{2}ln\eta_{1122}+4x_{1}x_{2}^{3}ln\eta_{2221}+x_{2}^{4}ln\eta_{2}-ln\left[x_{1}+x_{2}\left(M_{2}/M_{1}\right)\right]+\;4x_{1}^{3}x_{2}ln\left[\left(3+M_{2}/M_{1}\right)/4\right]+6x_{1}^{2}x_{2}^{2}ln\left[\left(1+M_{2}/M_{1}\right)/2\right]+\;4x_{1}x_{2}^{3}ln\left[\left(1+3M_{2}/M_{1}\right)/4\right]+x_{2}^{4}ln\left[M_{2}/M_{1}\right]\;$$

(11) $$ln\eta =x_{1}ln\eta_{1}+x_{2}ln\eta_{2}+x_{1}x_{2}\sum_{j=0}^{n}\left(\frac{A_{j}{\left(x_{1}-x_{2}\right)}^{j}}{T}\right)$$

Here, *η*, *η*_1_, and *η*_2_ denote the dynamic viscosities of the solutions; *x*_1_ and *x*_2_ represent the mole fractions; *M*_1_ and *M*_2_ signify the molecular weights; *V*, *V*_1_, and *V*_2_ refer to the molar volumes of the solutions and the pure component; *T* stands for the temperature; *R* is the gas constant; and *d*, *α*_12_, *α*_21_, *η*_1112_, *η*_1122_, *η*_2221_, *A*_0_, *A*_1_, *A*_2_, and *A*_3_ are variable parameters. These parameters were assessed using the Levenberg–Marquardt algorithm [53], and the mean absolute deviation (ADD%) between the observed and computed values was calculated using the following relation:

(12) $$ADD\%=\frac{100}{m}\sum_{i=1}^{m}{\left|\frac{X_{exp}-X_{cal}}{X_{exp}}\right|}_{i}$$

where *X* represents the values of the specified properties, and *m* denotes the number of experimental values. The variable parameters and ADD values are presented in Table S3.

The interaction parameter *d* provides an evaluation of the intermolecular interactions present among the components in liquid mixtures [54]. A positive value signifies strong interactions within the mixture, whereas a negative value indicates weak interactions. Therefore, positive values found in binary liquid solutions suggest that strong intermolecular interactions exist among the solution components. Additionally, the ADD values indicate that the models’ predictive capacity improves with a rise in the number of parameters that can be adjusted in the correlation equation. Thus, for the systems studied, the Jouyban–Acree model best correlates the experimental viscosity data.

### 2.2. Excess Properties

The excess properties, such as excess molar volume (*V*^E^), viscosity deviation (Δ*η*), and excess Gibbs activation energy (Δ*G*^#E^), were calculated from experimental density and viscosity data, according to the following equations:

(13) $$V^{E}=\frac{\left[x_{1}M_{1}+x_{2}M_{2}\right]}{\rho }-\left[\frac{x_{1}M_{1}}{\rho_{1}}+\frac{x_{2}M_{2}}{\rho_{2}}\right]$$

(14) $$\Delta \eta =\eta -(x_{1}\eta_{1}+x_{2}\eta_{2})$$

(15) $$∆G^{\neq E}=RT\left[ln\eta V-\left(x_{1}ln\eta_{1}V_{1}+x_{2}ln\eta_{2}V_{2}\right)\right]$$

The Redlich–Kister [59] (Equation (16)) and Hwang [60] (Equation (17)) equations were tested to correlate the excess properties:

(16) $$X^{E}=x_{1}x_{2}\sum_{k=0}^{3}a_{k}{\left(2x_{1}-1\right)}^{k}$$

(17) $$X^{E}=x_{1}x_{2}\left(A_{0}+A_{1}x_{1}^{3}+A_{2}x_{2}^{3}\right)$$

where *X*^E^ may represent *V*^E^, Δ*η*, or Δ*G*^#E^, whereas *a*_k_, *A*_0_, *A*_1_, and *A*_2_ denote the polynomial coefficients that were determined using the Levenberg–Marquardt algorithm [53].

**Figure 1:**
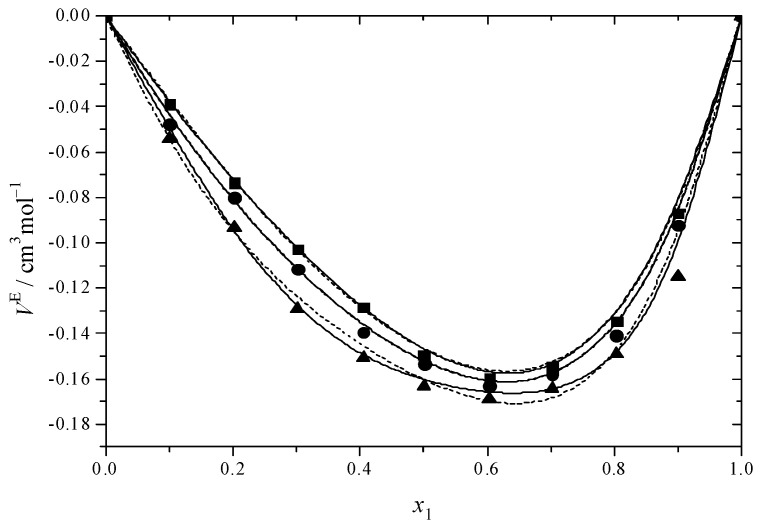
Excess molar volumes (*V*^E^) versus mole fraction for the DEGMEE (1) + MEA (2) system at ■ 293.15 K; ● 298.15 K; ▲ 303.15 K. The solid curves were determined with the R–K equation. The dash curves were determined with the Hwang equation.

**Figure 2:**
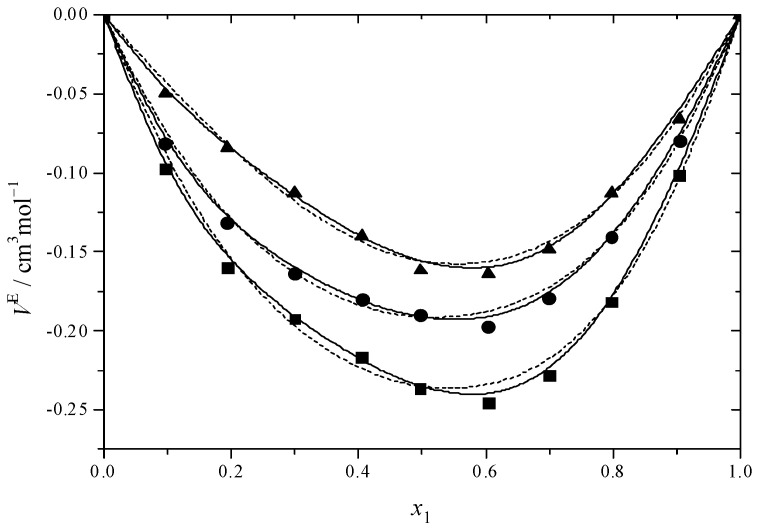
Excess molar volumes (*V*^E^) versus mole fraction for the MDEA (1) + MEA (2) system at ■ 293.15 K; ● 298.15 K; ▲ 303.15 K. The solid curves were determined with the R–K equation. The dash curves were determined with the Hwang equation.

Figure 1 and Figure 2 illustrate the results for the excess molar volume, which are presented additionally in Table S4. The values of *V*^E^ are negative for the systems under study.

In general, *V*^E^ values are influenced by intermolecular forces between the components of the mixture, by differences in shape and size, as well as by other intermolecular interactions such as induced forces, van der Waals forces, dispersion forces, or orientation forces [61]. In solutions with polar components, if the dipole–dipole or induced dipole–induced dipole interactions are disrupted during mixing, the induced force negatively affects *V*^E^. In solution systems lacking polar components, the dispersion force contributes and positively influences the *V*^E^ [62]. In the systems studied, the induced force significantly contributes to making *V*^E^ negative. The structural impacts resulting from the various shapes and sizes of molecules cause the mixture of molecules to adhere more closely, resulting in a negative *V*^E^ value. The molecular association additionally influences *V*^E^, with self-association demonstrating positive effects and intermolecular cross-association indicating negative effects [63]. The association of hydrogen bonds has been demonstrated to create network structures that decrease the volume of multi-component solutions [64]. In these binary systems, the intermolecular interactions primarily involve cross-association, with minimal or no self-association, as all *V*^E^ values are negative. The DEGMEE + MEA binary system presents intermolecular bonds of the –OH^…^… NH_2_ type that were formed between the N atom from the NH_2_ group in MEA and the H atom from the –OH group in DEGMEE. There are also strong intermolecular interactions of the –OH ^…^… O– type between the oxygen in the OH group of DEGMEE and the hydrogen in the OH group of MEA. The binary system MDEA + MEA presents intermolecular bonds formed between the N atom of MDEA and the H atom of the OH group of MEA.

In the binary systems, the *V*^E^ reaches its most negative value at *x* = 0.6, indicating that the intermolecular interactions between MEA with DEGMEE and MDEA are strong. Interactions among dissimilar molecules and structural factors involving the geometric modification of molecules according to size and shape variation, which can lead to a reduction in volume, were prevalent in binary mixtures at this value [65]. In comparison with the DEGMEE + MEA system, the *V*^E^ of the MDEA + MEA system is lower, due to the fact that the volume of MDEA is smaller than that of DEGMEE; thus, the filling effect is stronger. In addition, Figure 1 and Figure 2 show the dependence of *V*^E^ on temperature. For the DEGMEE + MEA system, the higher the temperature is, the more negative the *V*^E^ values become. However, for the MDEA + MEA system, the higher the temperature is, the less negative the *V*^E^ becomes. In the case of the DEGMEE + MEA system, the geometric arrangement contributes to the reduction of the volume of the binary mixtures in a greater proportion than the weakening of the hydrogen bonds; thus, with the increase in temperature, more negative values of *V*^E^ are obtained. For the MDEA + MEA system, when the temperature increases, the weakening of hydrogen bonds dominates the change of the excess molar volume in the mixing process; thus, less negative values of *V*^E^ are obtained.

The viscosity deviation of the mixtures represents, along with the excess volume, an important tool to describe the interactions present in the solutions. A negative value of the viscosity deviation of a binary solution shows that the real viscosity of the solution is lower than that of the ideal solution, while a positive viscosity deviation indicates that the viscosity of the real solution is higher than that of the ideal solution. When the interactions between the solution molecules are weak or intermolecular repulsion forces are present, the viscosity of the solution is lower. When strong intermolecular interactions, such as hydrogen bonds, van der Waals forces, or other intermolecular forces, are present, the viscosity of the mixture is higher. The Δ*η* values for the binary systems studied in this work are positive. The values of Δ*η*, whether positive or negative, rely on the equilibrium between two kinds of molecular interactions: self-association and cross-association. Self-association describes the affinity between similar molecules, which generally reduces the viscosity [22]. Cross-association denotes the attraction between various molecules, like hydrogen bonding and dipole–dipole interactions, which can enhance viscosity [66,67].

**Figure 3:**
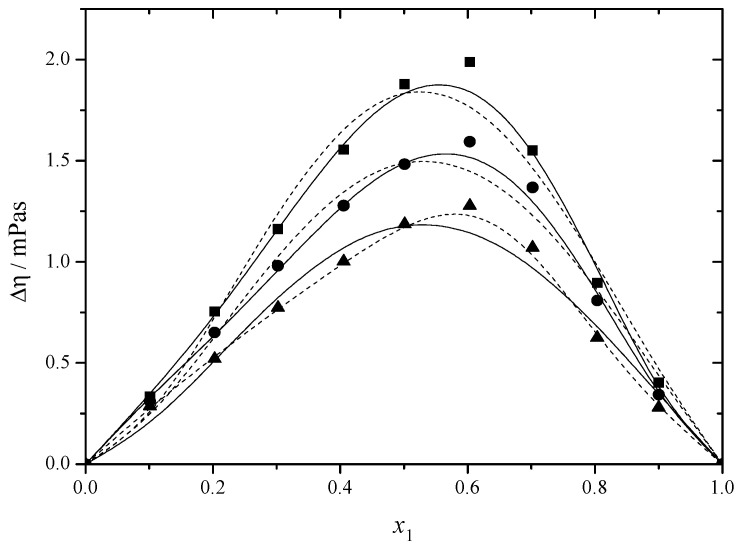
Viscosity deviation (Δ*η*) versus mole fraction for the DEGMEE (1) + MEA (2) system at ■ 293.15 K; ● 298.15 K; ▲ 303.15 K. The solid curves were determined with the R–K equation. The dash curves were determined with the Hwang equation.

**Figure 4:**
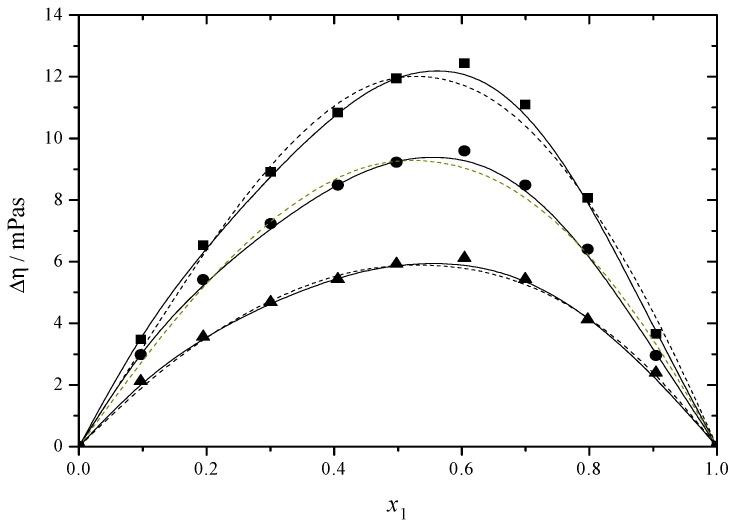
Viscosity deviation (Δ*η*) versus mole fraction for the MDEA (1) + MEA (2) system at ■ 293.15 K; ● 298.15 K; ▲ 303.15 K. The solid curves were determined with the R–K equation. The dash curves were determined with the Hwang equation.

Figure 3 and Figure 4 and Table S4 show the viscosity deviation values. The Δ*η* values of the DEGMEE + MEA and MDEA + MEA binary solutions are positive, indicating that the intense interactions among different molecules outweigh the impact of their self-association on the viscosity. The values drop at elevated temperatures since a rise in temperature leads to faster molecular motion, resulting in increased molecular kinetic energy [22].

Δ*G*^#E^ serves as a reliable parameter for assessing the molecular interactions within a system [68]. Positive Δ*G*^#E^ values indicate that there are significant and attractive interactions between various molecules, like hydrogen bonding. Nonetheless, negative values indicate a lack of such interactions and the dominance of interactions among identical molecules, like intermolecular dispersive forces, which may result in negative Δ*G*^#E^ values [69].

**Figure 5:**
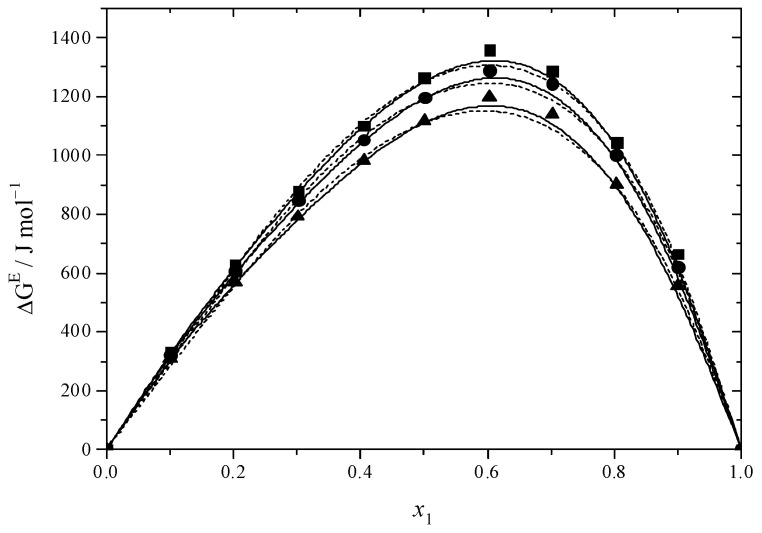
Excess Gibbs energy (Δ*G*^#E^) versus mole fraction for the DEGMEE (1) + MEA (2) system at ■ 293.15 K; ● 298.15 K; ▲ 303.15 K. The solid curves were determined with the R–K equation. The dash curves were determined with the Hwang equation.

**Figure 6:**
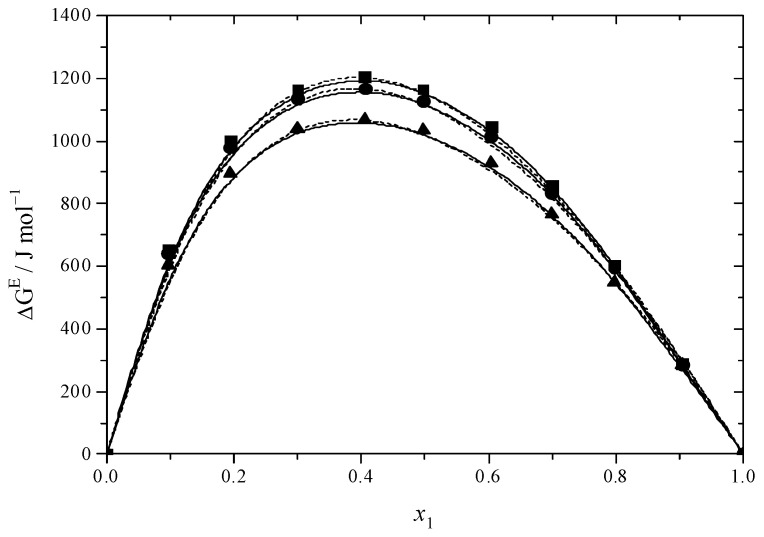
Excess Gibbs energy (*ΔG*^#E^) versus mole fraction for the MDEA (1) + MEA (2) system at ■ 293.15 K; ● 298.15 K; ▲ 303.15 K. The solid curves were determined with the R–K equation. The dash curves were determined with the Hwang equation.

The values obtained from Δ*G*^#E^ can be found in Figure 5 and Figure 6 as well as in Table S4. The Δ*G*^#E^ values for the DEGMEE + MEA and MDEA + MEA systems are positive, suggesting the existence of interactions between different molecules.

The parameters and standard deviation *σ* (Equation (7)) for *V*^E^, Δ*η*, and Δ*G*^#E^ determined using the Redlich–Kister and Hwang equations are presented in Table S5. The data shown indicate that the Redlich–Kister equation provides the best correlation for all excess properties in both systems.

### 2.3. Thermodynamic Functions of Activation

Equations (18) and (19) lead to the determination of the activation energies of the flow process [70]:

(18) $$\eta =\frac{hN}{V}exp\left(\frac{∆G^{\neq }}{RT}\right)$$

(19) $$∆G^{\neq }=∆H^{\neq }-T∆S^{\neq }$$

where *η* represents the viscosity of a solution; *h* signifies Planck’s constant; *N* denotes Avogadro’s number; *V* stands for the molar volume of the mixture; *R* is the universal gas constant; *T* indicates temperature; and $∆G^{\neq }$, $∆H^{\neq },$ and $∆S^{\neq }$ represent the molar Gibbs energy, enthalpy, and entropy of activation, respectively. The graphs of ln(*ηV*/*hN*) versus 1/*T* indicate a linear correlation, enabling the calculation of enthalpy ($∆H^{\neq }$) and entropy ($∆S^{\neq }$) of viscous flow based on the slopes and intercepts. The values of Gibbs activation energy ($∆G^{\neq }$) were also calculated and presented in Figures S5 and S6. Table 3 provides the values for the thermodynamic activation functions.

For binary systems, both $∆G^{\neq }$ and $∆H^{\neq }$ values are positive and decrease with rising DEGMEE concentration in the solution at a constant temperature for the DEGMEE + MEA system; however, in the case of the MDEA + MEA system, the values rise as the MDEA concentration increases. The values of $∆G^{\neq }$ at a constant concentration decrease with a rise in temperature. The values of $∆S^{\neq }$ for amines are almost five times higher than for DEGMEE. The values of $∆S^{\neq }$ are all positive for every compound and binary mixture, rising with the increase in MEA concentration in the DEGMEE + MEA system and with the Increase in MDEA concentration in the MDEA + MEA system.

### 2.4. FT-IR Spectra

**Figure 7:**
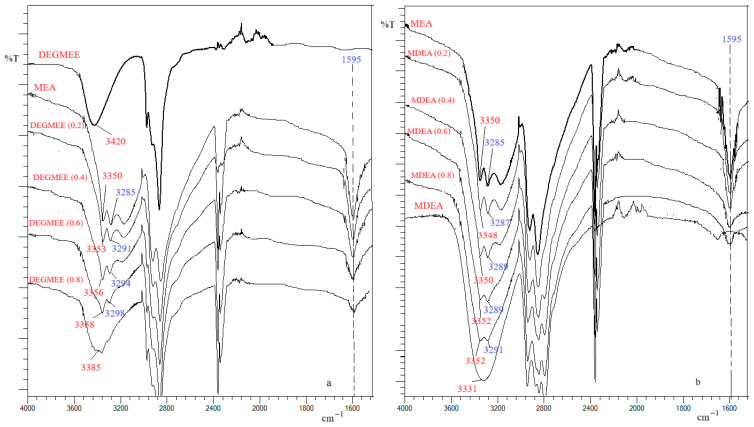
FT-IR spectra of the binary mixture at different mole fractions (*x*) and at room temperature: (**a**) DEGMEE (*x*) + MEA; (**b**) MDEA (*x*) + MEA.

IR spectrometry is an instrumental analysis method that allows the identification of the structure of organic compounds as well as the study of interactions between molecules of pure compounds and binary solutions of components. In this regard, an important place is occupied by the study of hydrogen bonds. The FT-IR spectra of the pure components (MEA, MDEA, and DEGMEE) and solutions with different concentrations were recorded in this work and are illustrated in Figure 7. The stretching vibration of the OH group shows two characteristic values, 3650–3590 cm^−1^ (sharp band) for free OH and 3550–3200 cm^−1^ (broad band) for OH involved in hydrogen bonds [17].

In Table S6, the bands and wave numbers (cm^−1^) of atom groups from pure compounds and binary mixtures are presented. The characteristic absorption bands at 3350, 3420, and 3331 cm^−1^ are due to the stretching vibrations of the O–H group in MEA, DEGMEE, and MDEA, respectively. The greater the difference between the frequency of the unassociated *v*OH bond and the associated *v*OH bond, the stronger the hydrogen bond [71]. This means that the strength of the hydrogen bond in the pure compounds analyzed varies as follows: MDEA > MEA > DEGMEE. For MEA solutions with DEGMME, it was observed that by increasing the MEA concentration in the solution, the stretching vibration band of the O–H group shifts to lower frequencies (for example, 3353 cm^−1^ for the solution with *x* = 0.2 DEGMEE). The shift of this absorption band to lower wavenumbers shows strong interaction between MEA molecules and DEGMEE molecules like hydrogen bonding between the oxygen in the OH group of DEGMEE with the hydrogen in the OH group of MEA. For the MEA + MDEA system, increasing the MEA concentration produces a small shift of the O–H stretching band to lower wavenumbers with the effect of slightly increasing the strength of the hydrogen bond formed between MEA and MDEA molecules. The stretching vibration band of the N–H atom group in MEA shifts slightly to lower wavenumbers as the MEA concentration in the MEA + DEGMEE and MEA + MDEA mixtures increases. This shift shows a slight weakening of the N–H bond probably caused by the formation of a hydrogen bond between the proton of the H–O group in the DEGMEE or MDEA molecule and the nitrogen atom in the MEA molecule. The H–N–H scissoring band, specific to MEA, appears at the same wave number (1595 cm^−1^) regardless of the MEA concentration in solutions with DEGMEE or with MDEA. The intensity and position of the bands of the IR spectra highlighted the presence of hydrogen bonds between the MEA + DEGMEE and MEA + MDEA molecules. These results support the negative values of the excess molar volume and the positive values of the viscosity deviation and of the excess Gibbs activation energy in both studied systems in this work.

### 2.5. CO_2_ Absorption

**Figure 8:**
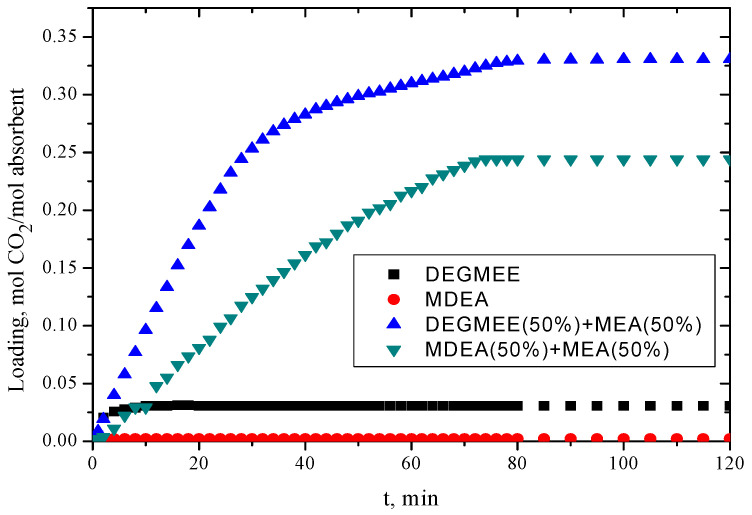
Loading CO_2_ of pure DEGMEE, pure MDEA, DEGMEE (50%) + MEA (50%), and MDEA (50%) + MEA (50%) binary systems.

The CO_2_ absorption capacities of 50 g DEGMEE, 50 g MDEA, 50 g binary mixture DEGMEE (50%) + MEA (50%), and 50 g binary mixture MDEA (50%) + MEA (50%) were determined experimentally, and the results are presented in Figure 8. From Figure 8, it is observed that pure DEGMEE and pure MDEA exhibit a significantly low efficiency for CO_2_ absorption. In the mixture of DEGMEE and MEA, the loading increased quickly from 0 to 40 min, indicating a rapid CO_2_ absorption. Up to 70 min, the absorption loading showed a slight increase, after which it is constant, suggesting saturation of the absorption. For the MDEA + MEA mixture, the loading of absorbent increased gradually until 70 min, at which point the loading remains constant. The CO_2_ absorption capacity for the DEGMEE + MEA mixture was around 0.3308 mol CO_2_ per mol of absorbent, whereas the MDEA + MEA mixture had a capacity of 0.2441 mol CO_2_ per mol of absorbent. It is noted that the addition of DEGMEE increases the absorption capacity of the DEGMEE + MEA mixture compared to the MDEA + MEA mixture. The incorporation of the DEGMEE to the amine forms a new hydrogen bond, which diminishes the volatilization of the mixture and decreases its vapor pressure [72].

## 3. Materials and Methods

### 3.1. Materials

Table 4 includes all details of chemical samples. All determinations were taken at a pressure of 100.5 kPa, which was determined in our laboratory with an accuracy of ±2 kPa.

### 3.2. Experimental Analysis

The samples were made by weighing with an Adventurer AX 224M analytical balance (Ohaus Corporation, Parsippany, NJ, USA), achieving a precision ±10^−4^ g. The uncertainty in the mole fraction of the solution was less than 0.0003. The densities of pure liquids and their combinations were assessed utilizing an digital densimeter (model KEM DA 650—Kyoto Electronics manufacturing, Tokyo, Japan) at *p* = 100.5 kPa. The temperature was recorded with an integrated thermometer along with a Peltier element. The producer indicated the repeatability for density and temperature measurements as 0.0002 g cm^−3^ and 0.1 K, respectively. The densimeter was calibrated using bidistilled and degassed water before and after every density measurement. The combined expanded uncertainty of the densities is assessed to be within 0.0007 g·cm^−3^. The estimated expanded uncertainties for the excess volume are 0.08 cm^3^·mol^−1^ (0.95 of confidence).

The viscosities of the pure compounds and binary mixtures were assessed using the Ubbelohde kinematic viscosity meter ViscoClock (Schott-Gerate GmbH, Mainz, Germany), which was maintained in a vertical position in a thermostatic bath (model TV 2000 Tamson, Bleiswijk, The Netherlands). The temperature was controlled to a precision of ±0.05 K.

The formula was utilized to determine the kinematic viscosity:

(20) $$\nu =At-\left(B/t\right)$$

were *ν* signifies the kinematic viscosity, *t* indicated the flow time, and *A* and *B* are specific constants of the viscometer. The constants *A* and *B* were established using doubly distilled water and benzene (Merck, Boston, MA, USA, mole fraction purity ≥ 0.995) as the calibrating liquids. The precision of time measurement is ±0.01 s.

Equation (21) was applied to determine the dynamic viscosity as follows:

(21) η=νρ

where *ρ* is the density.

The total expanded uncertainty for dynamic viscosity was determined to be 0.03 mPa.s. The expanded uncertainties in the ∆η were found to be 0.05 mPa.s at a confidence level of 0.95.

### 3.3. Spectral Analyses

FT-IR spectra were conducted at a pressure of 100.5 kPa and at ambient temperature. FT-IR spectra for DEGMEE (*x*) + MEA and MDEA (*x*)+ MEA binary mixtures at mole fractions of *x* = 0.0, 0.2, 0.4, 0.6, 0.8, and 1.0 were obtained using the IRAFINITY spectrometer (Schimatzu, Columbia, MD, USA), covering the wavelength range of 4000–500 cm^−1^.

### 3.4. CO_2_ Absorption

The CO_2_ absorption efficiency per mole of DEGMEE (1) + MEA (2) and MDEA (1) + MEA (2) binary mixtures was evaluated and compared with the pure compounds. A 50 g absorbent (pure DEGMEE, pure MDEA, DEGMEE + MEA and MDEA + MEA) was utilized to absorb 99.9% CO_2_. The absorbed CO_2_ quantity was weighted at every 2 min up to 80 min and then every 5 min until no CO_2_ absorption occurred, signifying that the absorbent was saturated. The flow rate of the gas was set to 100 mL per minute, and the determinations were made at room temperature (293.15 K) and a pressure of 100.5 kPa.

## 4. Conclusions

For the binary systems of DEGMEE (1) + MEA (2) and MDEA (1) + MEA (2), the densities and viscosities were experimentally determined at 293.15, 298.15, and 303.15 K and 100.5 kPa. The densities were correlated as a function of composition with the Belda and Herraez equations and as a function of composition and temperature with the Emmerling et al. and Gonzalez-Olmos–Iglesias equations. The best values of the standard deviation were obtained using the Herraez and Emmerling et al. equations. The experimental viscosity was correlated as a function of composition with four equations containing one, two, three, and four parameters. The four-parameter equation is the most suitable to correlate the experimental viscosity data. Based on the experimental density and viscosity values, the excess properties (excess volume, viscosity deviation, and excess Gibbs activation energy) were calculated and correlated with the Redlich–Kister and Hwang equations. The excess volume presents negative values for both studied systems, which indicates that there are strong intermolecular bonds in the solution. Positive values are obtained for the viscosity deviation and the excess Gibbs activation energy. The activation energies were calculated and discussed. The values of $∆G^{\neq }$ and $∆H^{\neq }$ are positive for both binary systems. The values of $∆S^{\neq }$ for amines are almost five times higher than for DEGMEE and are positive for every compound and binary solution. The FT-IR spectral analysis confirmed the type of interactions in the studied systems. The CO_2_ absorption capacities were determined for the pure components and for the mixtures of DEGMEE (50%) + MEA (50%) and MDEA (50%) + MEA (50%). It was found that the mixture formed by DEGMEE and MEA has a higher CO_2_ absorption capacity compared to the MDEA + MEA mixture.

## Figures and Tables

**Table 1:** Comparison of experimental values (*ρ* and *η*) of the pure components with the literature values at 293.15, 298.15, and 303.15 K.

Component	T/(K)	*ρ/*(g·cm^−3^)		*η*/(mPas)	
		This Work	Lit. Value	This Work	Lit. Value
MEA	293.15 298.15 303.15	1.0162 1.0125 1.0095	1.0158 [30] 1.0156 [31] 1.0161 [32] 1.0120 [31] 1.0124 [33] 1.0125 [34] 1.0091 [35] 1.0082 [31] 1.0084 [33]	23.77 18.65 14.79	23.54 [30] 23.66 [31] 23.65 [44] 18.64 [31] 18.88 [45] 14.60 [30] 15.04 [31] 14.75 [44]
DEGMEE	293.15 298.15 303.15	0.9890 0.9848 0.9805	0.9887 [36] 0.9885 [37] 0.9842 [36] 0.9846 [38] 0.9794 [36] 0.9791 [39]	4.59 3.89 3.41	4.551 [36] 3.861 [36] 3.368 [36]
MDEA	293.15 298.15 303.15	1.0402 1.0362 1.0325	1.0406 [40] 1.03966 [41] 1.03556 [42] 1.037863 [43] 1.0367 [25] 1.03213 [41] 1.0328 [40] 1.0329 [25]	102.92 74.93 57.58	102.7 [42] 103.3 [40] 74.81 [46] 75.90 [47] 57.57 [48] 57.615 [41] 57.3 [40]

Standard uncertainties: *u*(*p*) = 2 kPa and *u*(*T*) = 0.05 K; Expanded uncertainties: *U*(*ρ*) = 0.0007 g.cm^−3^ and *U*(*η*) = 0.03 mPa.s (0.95 of confidence).

**Table 2:** Density and viscosity data as a function of the mole fraction at 293.15, 298.15, and 303.15 K and *p* = 100.5 kPa.

*T*/(K)						
*x*_1_	*ρ*/(g·cm^−3^)			*η*/(mPa·s)		
	293.15	298.15	303.15	293.15	298.15	303.15
DEGMEE (1) + MEA (2)						
0.1013 0.2028 0.3022 0.4052 0.5011 0.6033 0.7020 0.8035 0.9000	1.0112 1.0072 1.0040 1.0011 0.9988 0.9966 0.9947 0.9928 0.9909	1.0076 1.0034 1.0001 0.9972 0.9948 0.9926 0.9906 0.9887 0.9868	1.0044 1.0002 0.9967 0.9936 0.9910 0.9886 0.9865 0.9846 0.9828	22.162 20.636 19.136 17.553 16.037 14.185 11.855 9.253 6.909	17.467 16.309 15.171 13.949 12.739 11.341 9.660 7.603 5.714	13.926 13.004 12.125 11.181 10.274 9.203 7.871 6.270 4.828
MDEA (1) + MEA (2)						
0.0970 0.1949 0.3006 0.4059 0.4978 0.6044 0.6995 0.7972 0.9041	1.0218 1.0261 1.0296 1.0325 1.0347 1.0368 1.0382 1.0392 1.0399	1.0178 1.0219 1.0254 1.0282 1.0303 1.0324 1.0338 1.0349 1.0358	1.0142 1.0180 1.0214 1.0243 1.0265 1.0285 1.0299 1.0310 1.0320	34.926 45.730 56.475 66.733 75.117 84.051 90.236 94.933 98.981	27.093 35.036 42.804 49.975 55.890 62.256 66.504 69.914 72.488	21.066 26.690 32.338 37.598 42.022 46.775 50.157 53.023 55.878

Standard uncertainties: *u*(*x*_1_) = 3 × 10^−4^, *u*(*p*) = 2 kPa, and *u*(*T*) = 0.05 K; Expanded uncertainties: *U*(*ρ*) = 0.0007 g.cm^−3^ and *U*(*η*) = 0.03 mPa.s (0.95 of confidence).

**Table 3:** Values of $∆G^{\neq }$, $∆H^{\neq }$, and $∆S^{\neq }$ for the binary mixtures.

*x*_1_	$∆H^{\neq }$ (kJ/mol)	$∆S^{\neq }$ (J/mol·K)	$∆G^{\neq }$ (kJ/mol) *T* (K)		
			293.15	298.15	303.15
DEGMEE (1) + MEA (2)					
0.0000 0.1013 0.2028 0.3022 0.4052 0.5011 0.6033 0.7020 0.8035 0.9000 1.0000	34.57 33.83 33.60 33.17 32.76 32.32 31.38 29.65 28.14 25.88 21.32	49.90 46.97 45.89 44.27 42.83 41.45 38.64 33.65 30.01 24.26 11.63	19.94 20.06 20.15 20.20 20.21 20.17 20.05 19.79 19.34 18.77 17.91	19.69 19.83 19.92 19.97 19.99 19.96 19.86 19.62 19.19 18.65 17.85	19.44 19.59 19.69 19.75 19.78 19.75 19.67 19.45 19.04 18.53 17.79
MDEA (1) + MEA (2)					
0.0000 0.0970 0.1949 0.3006 0.4059 0.4978 0.6044 0.6995 0.7972 0.9041 1.0000	34.57 36.81 39.20 40.60 41.80 42.33 42.71 42.80 42.45 41.69 42.38	49.90 53.63 58.88 61.27 63.39 63.72 63.55 62.83 60.80 57.43 59.06	19.94 21.09 21.93 22.64 23.22 23.65 24.08 24.38 24.63 24.86 25.07	19.69 20.82 21.64 22.33 22.90 23.33 23.76 24.07 24.33 24.57 24.77	19.44 20.55 21.35 22.03 22.58 23.01 23.44 23.75 24.02 24.29 24.48

**Table 4:** Specification of chemical compounds.

Chemical Name	Molecular Formula	Source	CAS	Mass Fraction Purity	Purification
MEA DEGMEE MDEA	C_2_H_7_NO C_6_H_14_O_3_ C_5_H_13_NO_2_	Sigma Aldrich Merck Darmstadt, Germany	141-43-5 111-90-0 105-59-9	≥99.0% ≥98.0% ≥98.0%	None None None

## Data Availability

Data are contained within the article and Supplementary Materials.

## Supplementary Materials

The following supporting information can be downloaded at: https://www.mdpi.com/article/10.3390/molecules30071523/s1, Figure S1: Density (*ρ*) versus mole fraction for the DEGMEE (1) + MEA (2) system at ■ 293.15 K; ● 298.15 K; ▲ 303.15 K; Figure S2: Density (*ρ*) versus mole fraction for the MDEA (1) + MEA (2) system at ■ 293.15 K; ● 298.15 K; ▲ 303.15 K; Figure S3: Viscosity (*η*) versus mole fraction for the DEGMEE (1) + MEA (2) system at ■ 293.15 K; ● 298.15 K; ▲ 303.15 K; Figure S4: Viscosity (*η*) versus mole fraction for the MDEA (1) + MEA (2) system at ■ 293.15 K; ● 298.15 K; ▲ 303.15 K; Figure S5. Energy Gibbs of activation viscous flow ($∆G^{\neq }$) versus mole fraction for the DEGMEE (1) + MEA (2) system at ■ 293.15 K; ● 298.15 K; ▲ 303.15 K; Figure S6: Energy Gibbs of activation viscous flow ($∆G^{\neq }$) versus mole fraction for the MDEA (1) + MEA (2) system at ■ 293.15 K; ● 298.15 K; ▲ 303.15 K; Table S1: Values of parameters at T = 293.15–303.15 K for the Belda and Herraez models and standard deviations (*σ*); Table S2: Values of parameters at T = 293.15–303.15 K for the Emmerling et al. and Gonzales-Olmos–Iglesias models and standard deviations (*σ*)^1^; Table S3: Values of parameters for the relations of Grunberg–Nissan, Heric–Brewer, four-body McAllister, and Jouyban–Acree and average absolute deviation at T = 293.15–303.15 K; Table S4: Excess molar volume (*V*^E^/cm^3^ mol^−1^), viscosity deviation (Δ*η*/mPa s), and excess Gibbs energy of activation of viscous flow (*ΔG*^#E^/J mol^−1^) of the DEGMEE (1) + MEA (2) and MDEA (1) + MEA (2) binary systems; Table S5: Polynomial coefficients and standard deviations (*σ*) for the binary systems; Table S6: Characteristic frequencies (cm^−1^) of pure compounds and binary mixtures of DEGMEE + MEA and MDEA + MEA.
