# Supplementary material for: Excess Properties, FT-IR Spectral Analysis, and CO2 Absorption Performance of Monoethanolamine with Diethylene Glycol Monoethyl Ether or Methyldiethanolamine Binary Solutions
Source: Molecules. 2025 Mar 29;30(7):1523. doi: 10.3390/molecules30071523 (PMC11990135; doi:10.3390/molecules30071523)
Supplement: Supplementary file 1 [file molecules-30-01523-s001.zip › molecules-3528196-supplementary.pdf]

Excess properties, FT-IR spectral analysis and CO_2 absorption performance of monoethanolamine with diethylene glycol monoethyl ether or methyldiethanolamine binary solutions

Maria Magdalena Naum [1,*], Mihaela Neagu [2,*] and Vasile Dumitrescu [1]

[1] Chemistry Department, Petroleum-Gas University of Ploiesti, 100680 Ploiesti, Romania; vdumi@upg-ploiesti.ro
[2] Petroleum Refining and Environmental Engineering Department, Petroleum-Gas University of Ploiesti, 100680 Ploiesti, Romania
* Correspondence: maria.budeanu@upg-ploiesti.ro (M.M.N.); mpetre@upg-ploiesti.ro (M.N.)

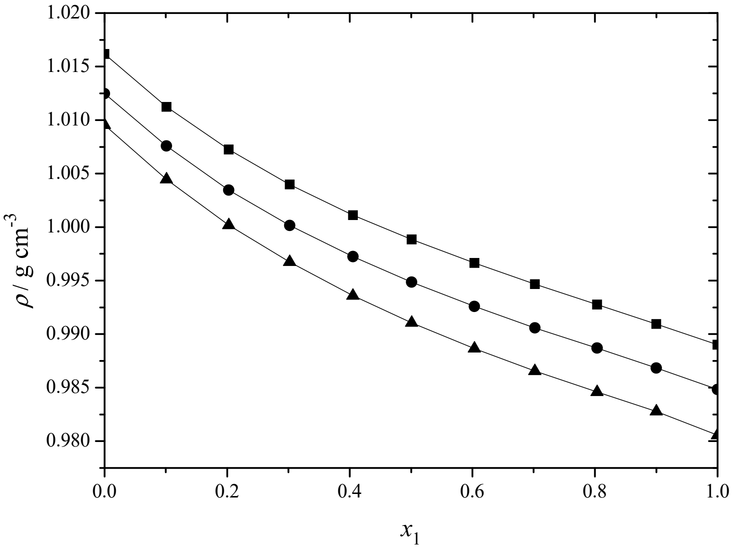

Figure S1. Density (ρ) versus mole fraction for DEGMEE (1) + MEA (2) system at: ■ 293.15 K; ● 298.15 K; ▲ 303.15 K.

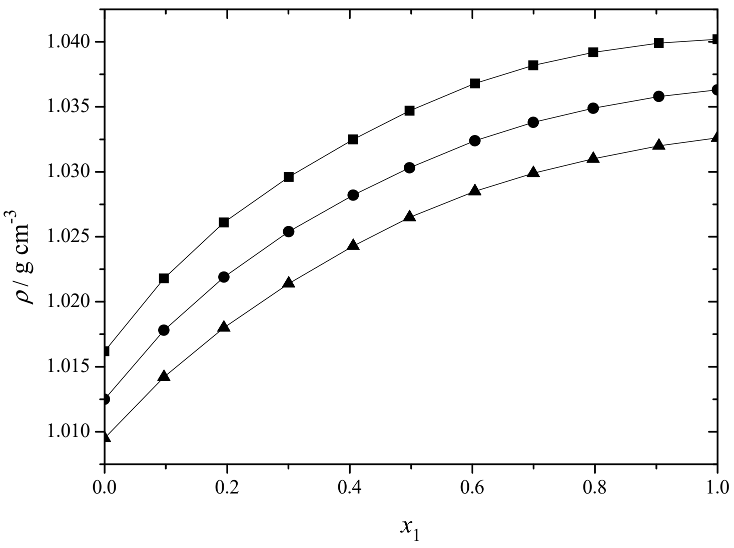

Figure S2. Density (ρ) versus mole fraction for MDEA (1) + MEA (2) system at: ■ 293.15 K; ● 298.15 K; ▲ 303.15 K.

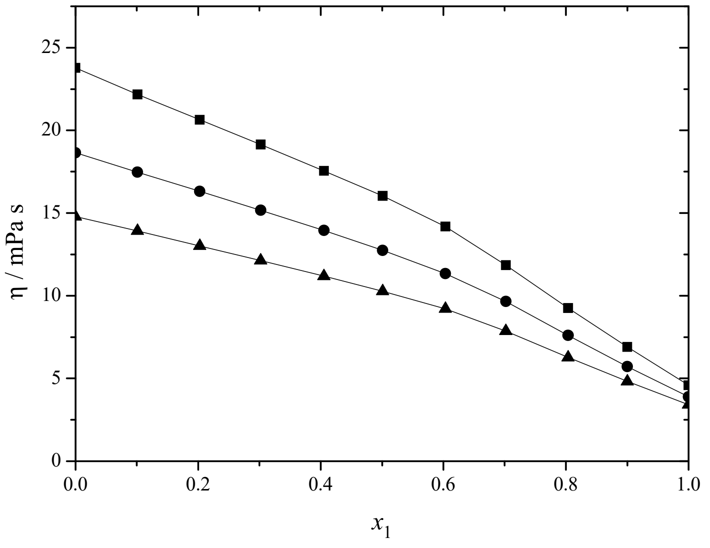

Figure S3. Viscosity (η) versus mole fraction for DEGMEE (1) + MEA (2) system at: ■ 293.15 K; ● 298.15 K; ▲ 303.15 K.

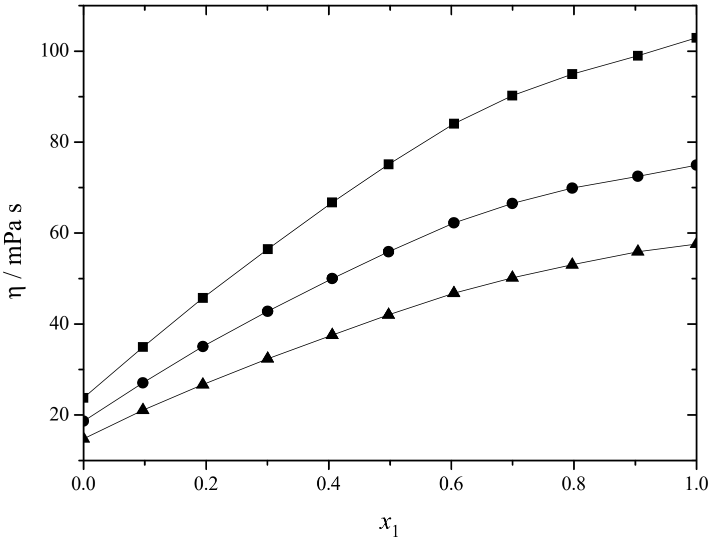

Figure S4. Viscosity (η) versus mole fraction for MDEA (1) + MEA (2) system at: ■ 293.15 K; ● 298.15 K; ▲ 303.15 K.

Table S1. Values of parameters at T = 293.15–303.15 K for the Belda and Herraez models and standard deviations (σ)

Equation	Parameters and σ	*T* (K)		
		293.15	298.15	303.15
DEGMEE (1) + MEA (2)				
Belda	m_1	−0.3491	−0.3375	−0.3116
	m_2	−0.6992	−0.6855	−0.6609
	$\sigma \cdot 10^4$	1.05	1.28	1.68
Herraez	*A*	0.7873	0.8016	0.8195
	B	−0.5370	−0.5785	−0.6283
	C	0.5100	0.5513	0.5925
	$\sigma \cdot 10^4$	0.40	0.46	0.66
MDEA (1) + MEA (2)				
Belda	m_1	0.4409	0.3190	0.4034
	m_2	−0.4325	−0.4643	−0.3681
	$\sigma \cdot 10^4$	1.68	1.15	0.62
Herraez	*A*	0.6742	0.6962	0.7479
	B	−0.6313	−0.6469	−0.7215
	C	0.0321	0.1418	0.2148
	$\sigma \cdot 10^4$	0.90	0.76	0.34

Table S2. Values of parameters at T = 293.15–303.15 K for the Emmerling *et al.* and Gonzales-Olmos-Iglesias models and standard deviations (σ)[1]

DEGMEE (1) + MEA (2)		
Emmerling *et al.*		
$A_1 = 1.0646$	$B_1 = 3.0665 \cdot 10^{-4}$	$C_1 = -1.9253 \cdot 10^{-6}$
$A_2 = 2.4993$	$B_2 = -0.0093$	$C_2 = 1.4498 \cdot 10^{-5}$
$P_1 = -0.9850$	$P_2 = 0.0066$	$P_3 = -1.1254 \cdot 10^{-5}$
$P_4 = 0.1594$	$P_5 = -9.6058 \cdot 10^{-4}$	$P_6 = 1.5297 \cdot 10^{-6}$
$P_7 = 0.8185$	$P_8 = -0.0057$	$P_9 = 9.8969 \cdot 10^{-6}$
	$10^4 \cdot \sigma = 0.44$	
Gonzalez-Olmos-Iglesias		
$Z_{00} = 2.5113$	$Z_{01} = -0.0094$	$Z_{02} = 1.4671 \cdot 10^{-5}$
$Z_{10} = -2.3967$	$Z_{11} = 0.0161$	$Z_{12} = -2.7456 \cdot 10^{-5}$
$Z_{20} = 0.9854$	$Z_{21} = -0.0066$	$Z_{22} = 1.1259 \cdot 10^{-5}$
	$10^4 \cdot \sigma = 5.00$	
MDEA (1) + MEA (2)		
Emmerling *et al.*		
$A_1 = 1.6205$	$B_1 = -0.0032$	$C_1 = 4.0385 \cdot 10^{-6}$
$A_2 = 2.5246$	$B_2 = -0.0095$	$C_2 = 1.4757 \cdot 10^{-5}$
$P_1 = 0.3495$	$P_2 = -0.0017$	$P_3 = 2.1730 \cdot 10^{-6}$
$P_4 = 4.3510$	$P_5 = -0.0294$	$P_6 = 4.9662 \cdot 10^{-5}$
$P_7 = -4.5633$	$P_8 = 0.0311$	$P_9 = -5.3009 \cdot 10^{-5}$
	$10^4 \cdot \sigma = 0.94$	
Gonzalez-Olmos-Iglesias		
$Z_{00} = 2.0798$	$Z_{01} = -0.0065$	$Z_{02} = 9.6542 \cdot 10^{-6}$
$Z_{10} = 0.0964$	$Z_{11} = 1.6500 \cdot 10^{-4}$	$Z_{12} = -1.1137 \cdot 10^{-6}$
$Z_{20} = -0.3786$	$Z_{21} = 0.0019$	$Z_{22} = -2.5063 \cdot 10^{-6}$
	$10^4 \cdot \sigma = 3.78$	

[1]Units: A_i, P_1, P_4, P_7, Z_{00}, Z_{10}, Z_{20}, σ: $g \cdot cm^{-3}$; B_i, P_2, P_5, P_8, Z_{01}, Z_{11}, Z_{21}: $g \cdot cm^{-3} K^{-1}$; C_i, P_3, P_6, P_9, Z_{02}, Z_{12}, Z_{22}: $g \cdot cm^{-3} K^{-2}$

Table S3. Values of parameters for the relations of Grunberg-Nissan, Heric-Brewer, four-body McAllister and Jouyban-Acree and average absolute deviation (*ADD*) at T = 293.15–303.15 K

Equation	Parameters and *ADD*	*T* (K)		
		293.15	298.15	303.15
DEGMEE (1) + MEA (2)				
Grunberg-Nissan	d	1.7573	1.6646	1.5183
	ADD	5.934	5.625	4.902
Heric-Brewer	α_{12}	2.0608	1.9682	1.8221
	α_{21}	0.9769	0.9182	0.7915
	ADD	0.618	0.658	0.580
McAllister	η_{1112}	14.8021	11.9079	9.3369
	η_{1122}	17.5665	13.8121	11.2987
	η_{2221}	20.3819	16.2307	12.9062
	ADD	0.326	0.384	0.462
Jouyban-Acree	A_0	505.5938	485.8979	453.1802
	A_1	328.3971	325.0391	299.9272
	A_2	63.8713	70.1216	47.1920
	A_3	−57.9881	−84.7960	−109.5686
	ADD	0.247	0.222	0.236
MDEA (1) + MEA (2)				
Grunberg-Nissan	d	1.7404	1.6864	1.5309
	ADD	4.469	4.376	3.920
Heric-Brewer	α_{12}	1.9639	1.9099	1.7542
	α_{21}	−0.8512	−0.8287	−0.7486
	ADD	1.157	1.257	1.286
McAllister	η_{1112}	99.9792	74.4494	56.9673
	η_{1122}	76.0174	55.2674	40.4859
	η_{2221}	72.8292	55.6563	41.5621
	ADD	0.641	0.630	0.560
Jouyban-Acree	A_0	493.0098	483.0053	442.5716
	A_1	−174.0704	−170.3336	−160.2518
	A_2	125.7331	144.1094	156.2620
	A_3	−183.4291	−186.2261	−154.9869
	ADD	0.074	0.066	0.23

Table S4. Excess molar volume (V^E / cm^{3}mol^{-1}), viscosity deviation ($\Delta\eta$ / mPa s) and excess Gibbs energy of activation of viscous flow ($\Delta G^{\#E}$ /J mol^{-1}) of DEGMEE (1) + MEA (2) and MDEA (1) + MEA (2) binary systems

	T / (K)								
	V^E /(cm^3·mol^{-1})			$\Delta\eta$ / (mPa·s)			$\Delta G^{\#E}$ / (J·mol^{-1})		
x_1	293.15	298.15	303.15	293.15	298.15	303.15	293.15	298.15	303.15
DEGMEE (1) + MEA (2)									
0.0000	0	0	0	0	0	0	0	0	0
0.1013	−0.039	−0.048	−0.054	0.334	0.313	0.287	330.65	322.08	310.26
0.2028	−0.073	−0.080	−0.094	0.755	0.651	0.521	627.72	606.16	570.11
0.3022	−0.103	−0.112	−0.129	1.161	0.981	0.773	880.01	846.92	792.31
0.4052	−0.128	−0.140	−0.150	1.554	1.278	1.002	1100.09	1052.52	980.54
0.5011	−0.149	−0.153	−0.163	1.878	1.483	1.187	1264.01	1196.85	1116.62
0.6033	−0.160	−0.163	−0.169	1.988	1.593	1.278	1358.59	1287.42	1197.29
0.7020	−0.155	−0.158	−0.164	1.551	1.368	1.071	1286.45	1242.92	1139.08
0.8035	−0.135	−0.141	−0.149	0.896	0.809	0.625	1044.61	1001.73	902.78
0.9000	−0.087	−0.092	−0.115	0.403	0.343	0.281	665.50	619.81	556.91
1.0000	0	0	0	0	0	0	0	0	0
MDEA (1) + MEA (2)									
0.0000	0	0	0	0	0	0	0	0	0
0.0970	−0.097	−0.081	−0.050	3.477	2.984	2.124	651.28	641.97	601.72
0.1949	−0.160	−0.131	−0.084	6.532	5.418	3.559	999.56	978.08	895.27
0.3006	−0.193	−0.164	−0.113	8.912	7.237	4.684	1163.09	1134.33	1039.27
0.4059	−0.217	−0.180	−0.140	10.835	8.483	5.438	1204.86	1166.07	1068.36
0.4978	−0.237	−0.190	−0.162	11.946	9.226	5.929	1163.77	1126.09	1033.65
0.6044	−0.245	−0.198	−0.164	12.443	9.592	6.121	1044.60	1015.43	929.62
0.6995	−0.228	−0.179	−0.148	11.102	8.489	5.433	858.19	834.20	765.08
0.7972	−0.182	−0.141	−0.113	8.066	6.400	4.118	605.10	597.07	549.20
0.9041	−0.101	−0.080	−0.066	3.654	2.958	2.400	287.07	284.45	284.91
1.0000	0	0	0	0	0	0	0	0	0

Standard uncertainties: $u(p)$ = 2 kPa, $u(T)$ = 0.05 K; Expanded uncertainties: $U(\rho)$ = 0.0007 g·cm^{-3}, $U(\eta)$ = 0.03 mPa·s, $U(V^E)$ = 0.08 cm^3·mol^{-1}, $U(\Delta\eta)$ = 0.05 mPa·s (0.95 of confidence).

Table S5. Polynomial coefficients and standard deviations (σ) for the binary systems

Equation	Parameters and σ	T / (K)		
		293.15	298.15	303.15
V^E / ($cm^3 \cdot mol^{-1}$)				
DEGMEE (1) + MEA (2)				
Redlich-Kister	a_0	−0.592	−0.613	−0.645
	a_1	−0.319	−0.274	−0.156
	a_2	−0.159	−0.229	−0.378
	a_3	−0.028	−0.091	−0.401
	σ	0.0011	0.0020	0.0035
Hwang	A	−0.539	−0.536	−0.518
	B	−0.604	−0.671	−0.860
	C	0.181	0.059	−0.152
	σ	0.0014	0.0019	0.0057
MDEA (1) + MEA (2)				
Redlich-Kister	a_0	−0.949	−0.772	−0.632
	a_1	−0.276	−0.146	−0.244
	a_2	−0.318	−0.238	0.004
	a_3	0.417	0.263	0.231
	σ	0.0026	0.0025	0.003
Hwang	A	−0.841	−0.692	−0.632
	B	−0.589	−0.388	−0.195
	C	−0.270	−0.255	0.199
	σ	0.007	0.0048	0.0053
$\Delta\eta$ / (mPa·s)				
DEGMEE (1) + MEA (2)				
Redlich-Kister	a_0	7.500	6.078	4.807
	a_1	3.513	3.148	2.593
	a_2	−5.940	−3.827	−2.899
	a_3	−5.619	−5.058	−4.550
	σ	0.060	0.035	0.031
Hwang	A	9.490	7.363	5.782
	B	−6.056	−3.441	−2.656
	C	−9.852	−6.825	−5.129
	σ	0.1075	0.0885	0.0785
MDEA (1) + MEA (2)				
Redlich-Kister	a_0	48.633	37.594	23.781
	a_1	17.141	10.939	5.758
	a_2	−9.404	−3.095	2.062
	a_3	−25.633	−17.618	−6.975
	σ	0.149	0.129	0.099
Hwang	A	51.670	38.559	23.066
	B	−2.179	1.983	6.881
	C	−22.157	−9.733	−1.172
	σ	0.449	0.314	0.153
$\Delta G^{\neq E}$ ($J \cdot mol^{-1}$)				
DEGMEE (1) + MEA (2)				
Redlich-Kister	a_0	5059.642	4824.726	4492.758
	a_1	2565.688	2491.964	2244.050
	a_2	601.329	641.997	442.179
	a_3	−510.199	−725.690	−923.745
	σ	11.00	8.43	8.84
	A	4861.253	4613.068	4347.880

Hwang	B	3655.745	3532.400	2887.500
	C	−2065.713	−1835.807	−1724.898
	σ	21.092	22.4208	24.0135
MDEA (1) + MEA (2)				
	a_0	4648.510	4500.759	4108.845
	a_1	−1577.679	−1519.869	−1418.644
Redlich-Kister	a_2	1076.690	1214.416	1302.735
	a_3	−1541.900	−1544.396	−1266.428
	σ	3.058	3.058	8.595
	A	4286.442	4092.715	3672.123
Hwang	B	−1072.948	−820.911	−470.840
	C	3968.163	4083.909	3963.580
	σ	14.13	14.42	13.47

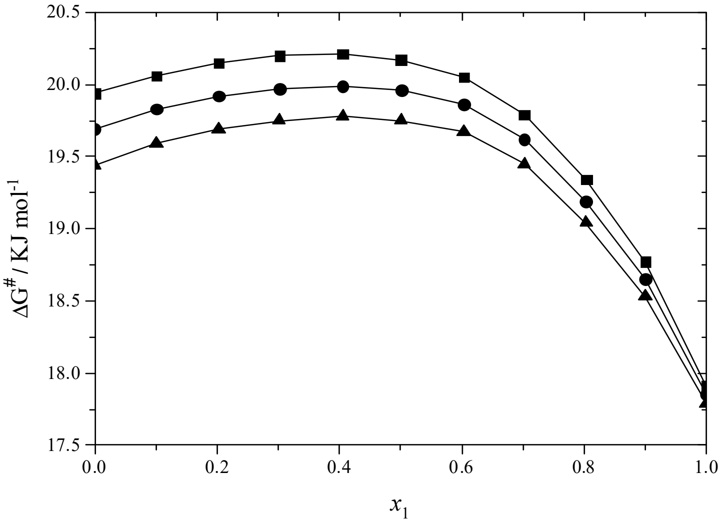

Figure S5. Energy Gibbs of activation viscous flow ($\Delta G^{\neq}$) versus mole fraction for DEGMEE (1) + MEA (2) system at: ■ 293.15 K; ● 298.15 K; ▲ 303.15 K.

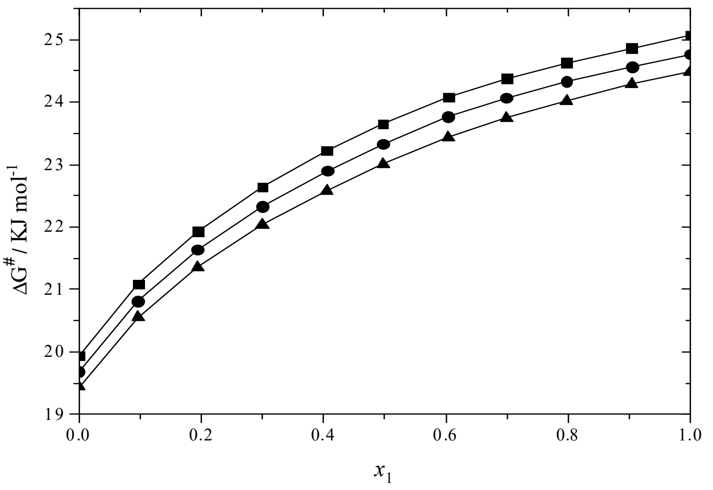

Figure S6. Energy Gibbs of activation viscous flow ($\Delta G^{\neq}$) versus mole fraction for MDEA (1) + MEA (2) system at: ■ 293.15 K; ● 298.15 K; ▲ 303.15 K.

Table S6. Characteristic frequencies (cm^{-1}) of pure compounds and binary mixtures of DEGMEE + MEA and MDEA + MEA

Compound	Band	Experimental $\upsilon/(cm^{-1})$
MEA	O–H stretching	3350
MEA	N–H stretching	3285
MEA	H–N–H scissoring	1595
DEGMEE	O–H stretching	3420
MDEA	O–H stretching	3331
MEA + DEGMEE (*x*=0.8)	O–H stretching	3385
MEA + DEGMEE (*x*=0.6)	O–H stretching	3358
MEA + DEGMEE (*x*=0.4)	O–H stretching	3356
MEA + DEGMEE (*x*=0.2)	O–H stretching	3353
MEA + DEGMEE (*x*=0.6)	N–H stretching	3298
MEA + DEGMEE (*x*=0.4)	N–H stretching	3294
MEA + DEGMEE (*x*=0.2)	N–H stretching	3291
MEA + DEGMEE (*x*=0.8)	H–N–H scissoring	1595
MEA + DEGMEE (*x*=0.6)	H–N–H scissoring	1595
MEA + DEGMEE (*x*=0.4)	H–N–H scissoring	1595
MEA + DEGMEE (*x*=0.2)	H–N–H scissoring	1595
MEA + MDEA (*x*=0.8)	O–H stretching	3352
MEA + MDEA (*x*=0.6)	O–H stretching	3352
MEA + MDEA (*x*=0.4)	O–H stretching	3350
MEA + MDEA (*x*=0.2)	O–H stretching	3348
MEA + MDEA (*x*=0.8)	N–H stretching	3291
MEA + MDEA (*x*=0.6)	N–H stretching	3289
MEA + MDEA (*x*=0.4)	N–H stretching	3289
MEA + MDEA (*x*=0.2)	N–H stretching	3287
MEA + MDEA (*x*=0.8)	H–N–H scissoring	1595
MEA + MDEA (*x*=0.6)	H–N–H scissoring	1595
MEA + MDEA (*x*=0.4)	H–N–H scissoring	1595
MEA + MDEA (*x*=0.2)	H–N–H scissoring	1595
